# Multiple sequence alignment with user-defined anchor points

**DOI:** 10.1186/1748-7188-1-6

**Published:** 2006-04-19

**Authors:** Burkhard Morgenstern, Sonja J Prohaska, Dirk Pöhler, Peter F Stadler

**Affiliations:** 1 Universität Göttingen, Institut für Mikrobiologie und Genetik, Abteilung für Bioinformatik, Goldschmidtstrasse. 1, D-37077 Göttingen, Germany; 2 Universität Leipzig, Institut für Informatik und Interdisziplinäres Zentrum für Bioinformatik, Kreuzstrasse 7b, D-04103 Leipzig, Germany

## Abstract

**Background:**

Automated software tools for multiple alignment often fail to produce biologically meaningful results. In such situations, expert knowledge can help to improve the quality of alignments.

**Results:**

Herein, we describe a *semi-automatic *version of the alignment program *DIALIGN *that can take pre-defined constraints into account. It is possible for the user to specify parts of the sequences that are assumed to be homologous and should therefore be aligned to each other. Our software program can use these sites as *anchor points *by creating a multiple alignment respecting these constraints. This way, our alignment method can produce alignments that are biologically more meaningful than alignments produced by fully automated procedures. As a demonstration of how our method works, we apply our approach to genomic sequences around the *Hox *gene cluster and to a set of DNA-binding proteins. As a by-product, we obtain insights about the performance of the *greedy *algorithm that our program uses for multiple alignment and about the underlying objective function. This information will be useful for the further development of DIALIGN. The described alignment approach has been integrated into the TRACKER software system.

## Background

Multiple sequence alignment is a crucial prerequisite for biological sequence data analysis, and a large number of multi-alignment programs have been developed during the last twenty years. Standard methods for multiple DNA or protein alignment are, for example, *CLUSTAL W *[1], *DIALIGN *[2] and *T-COFFEE *[3]; an overview about these tools and other established methods is given in [4]. Recently, some new alignment approaches have been developed such as *POA *[5], *MUSCLE *[6] or *PROBCONS *[7]. These programs are often superior to previously developed methods in terms of alignment quality and computational costs. The performance of multi-alignment tools has been studied extensively using various sets of real and simulated benchmark data [8-10].

All of the above mentioned alignment methods are fully *automated*, i.e., they construct alignments following a fixed set of algorithmical rules. Most methods use a well-defined *objective function *assigning numerical quality score to every possible output alignment of an input sequence set and try to find an optimal or near-optimal alignment according to this objective function. In this process, a number of program parameters such as gap penalties can be adjusted. While the overall influence of these parameters is quite obvious, there is usually no *direct *way of influencing the outcome of an alignment program.

Automated alignment methods are clearly necessary and useful where large amounts of data are to be processed or in situations where no additional expert information is available. However, if a researcher is familiar with a specific sequence family under study, he or she may already know certain parts of the sequences that are functionally, structurally or phylogenetically related and should therefore be aligned to each other. In situations where automated programs *fail *to align these regions correctly, it is desirable to have an alignment method that would accept such user-defined homology information and would then align the remainder of the sequences automatically, respecting these user-specified *constraints*.

The interactive program *MACAW *[11] can be used for semi-automatic alignment with user-defined constraints; similarly the program *OWEN *[12,13] accepts anchor points for pairwise alignment. Multiple-alignment methods accepting pre-defined constraints have also been proposed by Myers *et al*. [14] and Sammeth *et al*. [15]. The multi-alignment program DIALIGN [16,17] has an option that can be used to calculate alignments under user-specified constraints. Originally, this program feature has been introduced to reduce the alignment search space and program running time for large genomic sequences [18,19]; see also [20]. At *Göttingen Bioinformatics Compute Server (GOBICS)*, we provide a user-friendly web interface where anchor points can be used to guide the multiple alignment procedure [21]. Herein, we describe our anchored-alignment approach in detail using a previously introduced set-theoretical alignment concept. We apply our method to genomic sequences of the *Hox *gene clusters. For these sequences, the default version of DIALIGN produces serious mis-alignments where entire genes are incorrectly aligned, but meaningful alignments can be obtained if the known gene boundaries are used as anchor points.

In addition, our anchoring procedure can be used to obtain information for the further development of alignment algorithms. To improve the performance of automatic alignment methods, it is important to know what exactly goes wrong in those situations where these methods fail to produce biologically reasonable alignments. In principle, there are two possible reasons for failures of alignment programs. It is possible that the underlying *objective function *is 'wrong' by assigning high numerical scores to biologically meaningless alignments. But it is also possible that the objective function is 'correct' – i.e. biologically correct alignments have numerically optimal scores -and the employed heuristic *optimisation algorithm *fails to return mathematically optimal or near-optimal alignments. The anchoring approach that we implemented can help to find out which component of our alignment program is to blame if automatically produced alignments are biologically incorrect.

One result of our study is that anchor points can not only improve the *biological *quality of the output alignments but can in certain situations lead to alignments with significantly higher *numerical *scores. This demonstrates that the heuristic optimisation procedure used in DIALIGN may produce output alignments with scores far below the optimum for the respective data set. The latter result has important consequences for the further development of our alignment approach: it seems worthwile to develop more efficient algorithms for the optimisation problem that arises in the context of the DIALIGN algorithm. In other situations, the numerical scores of biologically correct alignments turned out to be below the scores of biololgically wrong alignments returned by the non-anchored version of our program. Here, improved optimisation functions will not lead to biologically more meaningful alignments. It is therefore also promising to develop improved objective function for our alignment approach.

## Alignment of tandem duplications

There are many situations where automated alignment procedures can produce biologically incorrect aligments. An obvious challenge are *distantly *related input sequences where homologies at the primary sequence level may be obscured by spurious random similarities. Another notorious challenge for alignment programs are *duplications *within the input sequences. Here, *tandem duplications *are particularly hard to align, see e.g. [22]. Specialised software tools have been developed to cope with the problems caused by sequence duplications [23]. For the segment-based alignment program DIALIGN, the situation is as follows. As described in previous publications, the program constructs pairwise and multiple alignments from pairwise local sequence similarities, so-called *fragment alignments or fragments *[17,16]. A fragment is defined as an un-gapped pair of equal-length segments from two of the input sequences. Based on statistical considerations, the program assigns a *weight score *to each possible fragment and tries to find a consistent collection of fragments with maximum total score. For pairwise alignment, a *chain *of fragments with maximum score can be identified [24]. For multiple sequence sets, all possible pairwise alignments are performed and fragments contained in these pairwise alignments are integrated *greedily *into a resulting multiple alignment.

**Figure 1:**
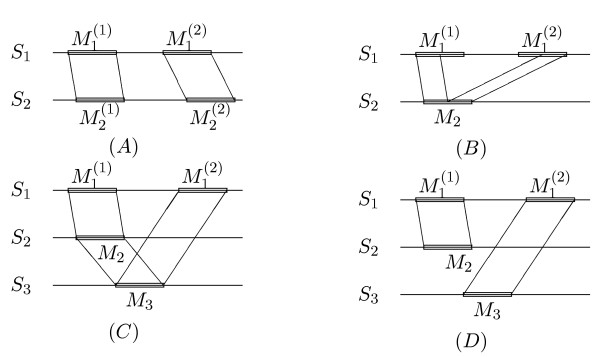
Possible mis-alignments caused by tandem duplications in the segment-based alignment approach (DIALIGN). We assume that various instances of a motif are contained in the input sequence set and that the degree of similarity among the different instances is approximately equal. For simplicity, we also assume that the sequences do not share any similarity outside the conserved motif. Lines connecting the sequences denote fragments identified by DIALIGN in the respective pairwise alignment procedures. (*A*) If a tandem duplication occurs in two sequences, the correct alignment will be found since the algorithm identifies a *chain *of local alignments with maximum *total *score. (*B*) If a motif is duplicated in one sequence but only one instance *M*_2 _is contained in the second sequence, it may happen that *M*_2 _is split up and aligned to different instances of the motif in the first sequence. (*C*) If the motif is duplicated in the first sequence but only one instance of it is contained in sequences two and three, respectively, *consistency *conflicts can occur. In this case, local similarities identified in the respective pairwise alignments cannot be integrated into one single output alignment. To select a consistent subset of these pairwise similarities, DIALIGN uses a *greedy *heuristic. Depending on the degree of similarity among the instances of the motif, the greedy approach may lead to serious mis-alignments (*D*).

As indicated in Figure 1, tandem duplications can create various problems for the above outlined alignment approach. In the following, we discuss two simple examples where duplications can confuse the segment-based alignment algorithm. Let us consider a motif that is duplicated in one or several of the input sequences *S*_1_,..., *S*_*k*_. For simplicity, let us assume that our sequences do not share any significant similarity outside the motif. Moreover, we assume that the degree of similarity among all instances of the motif is roughly comparable. There are no difficulties if two sequences are to be aligned and the motif is duplicated in *both *sequences, i.e if one has instances  and  of the motif in sequence *S*_1 _and instances  and  of the same motif in sequence *S*_2_ as in Figure 1 (A). In such a situation, our alignment approach will correctly align  to  and  to  since, for pairwise alignment, our algorithm returns a *chain *of fragments with maximum *total *score.

Note that a strictly greedy algorithm could be confused by this situation and could align, for example,  to  in Figure 1 if the similarity among these two instances of the motif happens to be slightly stronger than the similarity among  and , and among  and , respectively. However, DIALIGN uses a greedy approach only for *multiple *alignment where an exact solution is not feasible, but for pairwise alignment, the program returns an *optimal *alignment with respect to the underlying objective function. Thus, under the above assumtion, a meaningful alignment will be produced even if  exhibits stronger similarity to  than to .

The trouble starts if a tandem duplication ,  occurs in *S*_1 _but only one instance of the motif, *M*_2_, is present in *S*_2_. Here, it can happen that the beginning of *M*_2 _is aligned to the beginning of  and the end of *M*_2 _is aligned to the end of  as in Figure 1 (B). DIALIGN is particularly susceptible to this type of errors since it does not use gap penalties. The situation is even more problematic for multiple alignment. Consider, for example, the three sequences *S*_1_, *S*_1_, *S*_3 _in Figure 1 (C), where two instances ,  of a motif occur in *S*_1 _while *S*_2 _and *S*_3 _each contain only one instance of the motif *M*_2 _and *M*_3_, respectively. Under the above assumptions, a *biologically *meaningful alignment of these sequences would certainly align *S*_2 _to *S*_3_, and both motifs would be aligned either to  or to  – depending on the degree of similarity of *S*_2 _and *S*_3 _to  and , respectively. Note that such an alignment would also receive a high *numerical *score since it would involve *three *pairwise alignments of the conserved motif. However, since the pairwise alignments are carried out independently for each sequence pair, it may happen that the first instance of the motif in sequence *S*_1_,  is aligned to *M*_2 _but the second instance, , is aligned to *M*_3 _in the respective pairwise alignments as in Figure 1 (C). Thus, the correct alignment of *M*_2 _and *M*_3 _will be *inconsistent *with the first two pairwise alignments. Depending on the degree of similarity among the motifs, alignment of  and *M*_3 _may be rejected in the greedy algorithm, so these motifs may not be aligned in the resulting multiple alignment. It is easy to see that the resulting multiple alignment would not only be biologically questionable, but it would also obtain a numerically lower score as it would involve only *two *pairwise alignments of the motif.

## Multiple alignment with user-defined anchor points

To overcome the above mentioned difficulties, and to deal with other situations that cause problems for alignment programs, we implemented a semi-automatic *anchored *alignment approach. Here, the user can specify an arbitrary number of *anchoring points *in order to guide the alignment procedure. Each anchor point consists of a pair of equal-length segments of two of the input sequences. An anchor point is therefore characterised by five coordinates: the two *sequences *involved, the *starting positions *in these sequences and the *length *of the anchored segments. As a sixth parameter, our method requires a *score *that determines the *priority *of an anchor point. The latter parameter is necessary, since it is in general not meaningful to use *all *anchors proposed by the user. It is possible that the selected anchor points are *inconsistent *with each other in the sense that they cannot be included in one single multiple output alignment, see [16] for our concept of consistency. Thus, it may be necessary for the algorithm to select a suitable *subset *of the proposed anchor points.

Our software provides two slightly different options for using anchor points. There is a *strong *anchoring option, where the specified anchor positions are necessarily aligned to each other, consistency provided. The remainder of the sequences is then aligned based on the consistency constraints given by these pre-aligned positions. This option can be used to enforce correct alignment of those parts of the sequences for which additional expert information is available. For example, we are planning to align RNA sequences by using both primary and secondary structure information. Here, locally conserved secondary structures could be used as 'strong' anchor points to make sure that these structures are properly aligned, even if they share no similarity at the primary-structure level.

In addition, we have a *weak *anchoring option, where consistent anchor points are only used to constraint the output alignment, but are not necessarily aligned to each other. More precisely, if a position *x *in sequence *S*_*i *_is *anchored *with a position *y *in sequence *S*_*j *_through one of the anchor points, this means that *y *is the *only *position from *S*_*j *_that can be aligned to *x*. Whether or not *x *and *y *will actually appear in the same column of the output alignment depends on the degree of local similarity among the sequences around positions *x *and *y*. If no statistically significant similarity can be detected, *x *and *y *may remain un-aligned. Moreover, anchoring *x *and *y *means that positions strictly to the left (or strictly to the right) of *x *in *S*_*i *_can be aligned only to positions strictly to the left (or strictly to the right) of *y *in *S*_*j *_– and vice versa. Obviously, these relations are *transitive*, so if position *x *is anchored with position *y*_1_, *y*_1 _is to the left of another position *y*_2 _in the same sequence, and *y*_2 _in turn, is aligned to a position *z*, then positions to the left of *x *can be aligned only to positions to the left of *z *etc. The 'weak' option may be useful if anchor points are used to reduce the program running time.

Algorithmically, strong or weak anchor points are treated by DIALIGN in the same way as *fragments *( = segment pairs) in the greedy procedure for multi-alignment. By transitivity, a set *Anc* of anchor points defines a *quasi partial order relation *≤_*Anc *_on the set *X *of all positions of the input sequences – in exactly the same way as an alignment *Ali* induces a quasi partial order relation ≤_*Ali *_on *X *as described in [16,25]. Formally, we consider an alignment *Ali* as well as a set of anchor points *Anc* as an *equivalence relation *defined on the set *X *of all positions of the input sequences. Next, we consider the partial order relation ≤ on *X *that is given by the 'natural' ordering of positions within the sequences. In order-theoretical terms, ≤ is the *direct sum *of the *linear *order relations defined on the individual sequences. The partial order relation ≤_*Anc *_is then defined as the *transitive closure *of the union ≤ ∪ *Anc*. In other words, we have *x *≤_*Anc *_*y *if and only if there is a chain x_0_, ..., *x*_*k *_of positions with *x*_0 _= *x *and *x*_*k *_= *y *such that for every *i *∈ {1,..., *k*}, position *x*_*i*-1 _is either anchored with *x*_*i *_or *x*_*i*-1 _and *x*_*i *_belong to the same sequence, and *x*_*i*-1 _is on the left-hand side of *x*_*i *_in that sequence.

In our set-theoretical setting, a relation *R *on *X *is called consistent if all restrictions of the tansitive closure of the union ≤ ∪ *R *to the idividual sequences *coincides *with their respective 'natural' linear orderings. With the *weak *version of our anchored-alignment approach, we are looking for an alignment *Ali* wich maximum score such that the union *Ali* ∪ *Anc* is consistent. With the *strong *option, we are looking for a maximum-scoring alignment *Ali* that is a superset of *Anc*. With both program options, our optimisation problem is to find an alignment *Ali* with maximum score – under the additional constraint that the set-theoretical union *Ali* ∪ *Anc* is consistent. In the weak anchoring approach, the output alignment is *Ali* while with the strong option, the program returns the transitive closure of the union *Ali* ∪ *Anc*.

The above optimisation problem makes sense only if the set *Anc* of anchor points is itself consistent. Since a user-defined set of anchor points cannot be expectd to be consistent, the first step in our anchoring procedure is to select a consistent *subset *of the anchor points proposed by the user. To this end, the program uses the same greedy approach that it applies in the optimisation procedure for multiple alignment. That is, each anchor point is associated with some user-defined score, and the program accepts input anchor points in order of decreasing scores – provided they are consistent with the previously accepted anchors.

The greedy selection of anchor points makes it possible for the user to *prioritise *potential anchor points according to arbitrary user-defined criteria. For example, one may use known gene boundaries in genomic sequences to define anchor points as we did in the *Hox *gene example described below. In addition, one may want to use *automatically *produced local alignments as anchor points to speed up the alignment procedure as outlined in [18]. Note that the set of gene boundaries will be necessarily consistent as long as the relative ordering among the genes is conserved. However, the automatically created anchor points may well be *inconsistent *with those 'biologically defined' anchors or inconsistent with each other. Since anchor points derived from expert knowledge should be more reliable than anchor points identified by some software program, it would make sense to first accept the known gene boundaries as anchors and then to use the automatically created local alignments, under the condition that they are consistent with the known gene boundaries. So in this case, one could use local alignment scores as scores for the *automatically *created anchor points, while one would assign arbitrarily defined higher scores to the *biologically *verified gene boundaries.

## Applications to *Hox *gene clusters

As explained above, tandem duplications pose a hard problem for automatic alignment algorithms. Clusters of such paralogous genes are therefore particularly hard to align. As a real-life example we consider here the *Hox *gene clusters of vertebrates. *Hox *genes code for homeodomain transcription factors that regulate the anterior/posterior patterning in most bilaterian animals [26,27]. This group of genes, together with the so-called *ParaHox *genes, arose early in metazoan history from a single ancestral *"UrHox *gene" [28]. Their early evolution was dominated by a series of tandem duplications. As a consequence, most bilaterians share at least eight distinct types (in arthropods, and 13 or 14 in chordates), usually referred to as paralogy classes. These *Hox *genes are usually organised in tightly linked clusters such that the genes at the 5'end (paralogy groups 9–13) determine features at the posterior part of the animal while the genes at the 3'end (paralogy groups 1–3) determine the anterior patterns.

In contrast to all known invertebrates, all vertebrate lineages investigated so far exhibit multiple copies of *Hox *clusters that presumably arose through genome duplications in early vertebrate evolution and later in the actinopterygian (ray finned fish) lineage [29-33]. These duplication events were followed by massive loss of the duplicated genes in different lineages, see e.g. [34] for a recent review on the situation in teleost fishes. The individual *Hox *clusters of gnathostomes have a length of some 100,000nt and share besides a set of homologous genes also a substantial amount of conserved non-coding DNA [35] that predominantly consists of transcription factor binding sites. Most recently, however, some of these "phylogenetic footprints" were identified as microRNAs [36].

**Figure 2:**
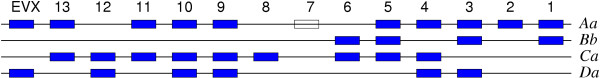
The pufferfish *Takifugu rubripes *has seven *Hox *clusters of which we use four in our computational example. The *Evx *gene, another homedomain transcription factor is usually liked with the *Hox *genes and can be considered as part of the *Hox *cluster. The paralogy groups are indicated. Filled boxes indicates intact *Hox *genes, the open box indicates a *HoxA7a *pseudogene [45].

**Figure 3:**
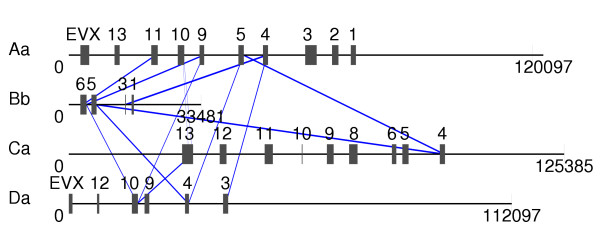
Result of a DIALIGN run on the *Hox *sequences from Figure 2 without anchoring. The diagram represents sequences and gene positions to scale. All incorrectly aligned segments (defined as parts of a gene that are aligned with parts of gene from a different paralogy group) are indicated by lines between the sequences.

Figure 2 and 3 show four of the seven *Hox *clusters of the pufferfish *Takifugu rubripes*. Despite the fact that the *Hox *genes within a paralogy group are significantly more similar to each other than to members of other paralogy groups, there are several features that make this dataset particularly difficult and tend to mislead automatic alignment procedures: (1) Neither one of the 13 *Hox *paralogy groups nor the *Evx *gene is present in all four sequences. (2) Two genes, *HoxC8a *and *HoxA2a *are present in only a single sequence. (3) The clusters have different sizes and numbers of genes (33481 nt to 125385 nt, 4 to 10 genes).

We observe that without anchoring DIALIGN mis-aligns many of of the *Hox *genes in this example by matching blocks from one *Hox *gene with parts of a *Hox *gene from a different paralogy group. As a consequence, genes that should be aligned, such as *HoxA1Oa *and *HoxDIOa*, are not aligned with each other.

Anchoring the alignment, maybe surprisingly, increases the number of columns that contain aligned sequence positions from 3870 to 4960, i.e., by about 28%, see Table 2. At the same time, the CPU time is reduced by almost a factor of 3.

We investigated not only the *biological *quality of the anchored and non-anchored alignments but also looked at their *numerical *scores. Note that in DIALIGN, the score of an alignment is defined as the sum of weight scores of the fragments it is composed of [17]. For some sequence sets we found that the score of the anchored alignment was above the non-anchored alignment while for other sequences, the non-anchored score exceeded the anchored one. For example, with the sequence set shown in Figure 2, the alignment score of the – biologically more meaningful – anchored alignment was > 13% *below *the non-anchored alignment (see Table 1). In contrast, another sequence set with five *HoxA *cluster sequences (TrAa, TnAa, DrAb, TrAb, TnAb) from three teleost fishes *(Takifugu rubripes*, Tr; *Tetraodon nigroviridis*, Tn; *Danio rerio*, Dr) yields an anchored alignment score that is some 15% *above *the non-anchored score.

**Table 1:** Effect of different anchors in the Fugu example of Figure 2. We consider aligned sequence positions in intergenic regions (i.e., *outside *the coding regions and introns) only. Column 2 gives the number of sequence positions for which DIALIGN added at least one additional sequence that was not represented in original TRACKER footprint. Column 3 lists the total number of nucleotides in footprints that were not detected by tracker but were aligned by anchored DIALIGN.

anchor	nt positions in footprints		
	total	expanding	new
none	1546	0	618
genes	1686	39	694
genes and BLASTZ hits	2433	39	841

**Table 2:** Aligned sequence positions that result from fragment aligments in the Fugu *Hox *cluster example. To compare these alignments, we counted the number of columns where two, three or four residues are aligned, respectively. Here, we counted only upper-case residues in the DIALIGN output since lower-case residues are not considered to be aligned by DIALIGN. The number of columns in which two or three residues are aligned increases when more anchors are used, while the number of columns in which all sequences are aligned decreases. This is because in our example no single *Hox *gene is contained in all four input sequences, see Figure 2. Therefore a biologically correct alignment of these sequences should not contain columns with four residues. CPU times are measured on a PC with two Intel Xeon 2.4GHz processors and 1 Gbyte of RAM.

anchor	alignment length	aligned sequences			CPU time	score
		2	3	4		
none	281759	2958	668	244	4:22:07	1166
genes	252346	3674	1091	195	1:18:12	1007
BLASTZ hits	239326	4036	1139	33	0:19:32	742

## Anchored protein alignments

BAliBASE is a benchmark database to evaluate the performance of software programs for multiple protein alignment [37]. The database consists of a large number of protein families with known 3D structure. These structures are used to define so-called *core blocks *for which 'biologically correct' alignments are known. There are two scoring systems to evaluate the accuracy of multiple alignments on BAliBASE protein families. The BAliBASE *sum-of-pairs *score measures the percentage of correctly aligned pairs of amino acid residues within the core blocks. By contrast, the *column score *measures the percentage of correctly aligned columns in the core blocks, see [38,10] for more details. These BAliBASE scoring functions are not to be confused with the objective functions used by different alignment algorithms.

Thus, alignment programs can be evaluated by their ability to correctly align these core blocks. BAliBASE covers various alignment situations, e.g. protein families with global similarity or protein families with large internal or terminal insertions or deletions. However, it is important to mention that most sequences in the standard version of BAliBASE are *not *real-world sequences, but have been artificially truncated by the database authors who simply removed non-homologous C-terminal or N-terminal parts of the sequences. Only the most recent version of BAliBASE provides the original full-length sequence sets together with the previous truncated data. Therefore, most studies based on BAliBASE have a strong bias in favour of *global *alignment programs such as CLUSTAL W [1]; these programs perform much better on the BAliBASE data than they would perform on on realistic full-length protein sequences. The performance of programs that are based on *local *sequence similarities, on the other hand, is systematically *underestimated *by BAliBASE. Despite this systematic error, test runs on BAliBASE can give a rough impression about the performance of multiple-alignment programs in different situations.

DIALIGN has been shown to perform well on those data sets in BAliBASE that contain large insertions and deletions. On the other hand, it is often outperformed by global alignment methods on those data sets where homology extends over the entire sequence length but similarity is low at the primary-sequence level. For the further development and improvement of the program, it is crucial to find out which components of DIALIGN are to blame for the inferiority of the program on this type if sequence families. One possibility is that biologically meaningful alignments on BAliBASE would have high numerical scores, but the greedy heuristic used by DIALIGN is inefficient and returns low-scoring alignments that do not align the core blocs correctly. In this case, one would use more efficient optimisation strategies to improve the performance of DIALIGN on BAliBASE. On the other hand, it is possible that the scoring function used in DIALIGN assigns highest scores to biologically wrong alignments. In this case, an improved optimisation algorithm would not lead to any improvement in the biological quality of the output alignments and it would be necessary to improve the objective function used by the program.

To find out which component of DIALIGN is to blame for its unsatisfactory performance on some of the BAliBASE data, we applied our program to BAliBASE (*a*) using the non-anchored default version of the program and (*b*) using the *core blocks *as anchor points in order to *enforce *biologically correct alignments of the sequences. We then compared the numerical DIALIGN scores of the anchored alignments to the non-anchored default alignments. The results of these program runs are summarised in Table 3. The numerical alignment scores of the (biologically correct) anchored alignments turned out to be slightly *below *the scores of the non-anchored default alignments.

**Table 3:** DIALIGN alignment scores for anchored and non-anchored alignment of five reference test sets from BAliBASE. As anchor points, we used the so-called *core-blocks *in BAliBASE, thereby enforcing biologically correct alignments of the input sequences. The figures in the first and second line refer to the sum of DIALIGN alignment scores of all protein families in the respective reference set. Line four contains the number of sequence sets where the anchoring *improved *the alignment score together with the total number of sequence sets in this reference set. Our test runs show that on these test data, biologically meaningful alignments do not have higher DIALIGN scores than alignments produced by the default version of our program.

	Alignment scores					
	Ref1	Ref2	Ref3	Ref4	Ref5	Total
non-anchored	53,613	269,009	283,273	36,515	29,214	671,624
anchored	53,417	265,966	283,136	36,611	29,257	668,387
ratio	0.996	0.988	0.999	1.002	1.001	0.995
score improved	23/82	13/23	4/23	6/16	4/12	50/156

**Figure 4:**
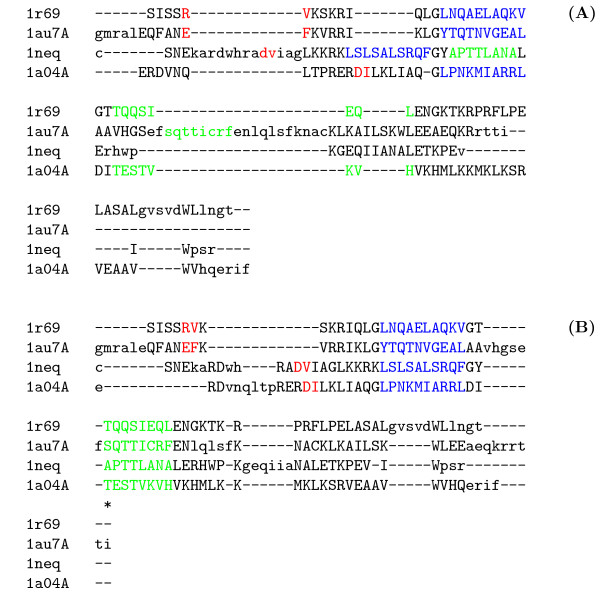
Anchored and non-anchored alignment of a set of protein sequences with known 3D structure (data set lr69 from BAliBASE [38]). Three *core blocks *for which the 'correct' alignment is known are shown in red, blue and green. **(A) **Alignment calculated by DIALIGN with default options. Most of the core blocks are mis-aligned. **(B) **Alignment calculated by DIALIGN with *anchoring *option. The first position of the third block has been used as anchor point, i.e. the program has *been forced *to align this column correctly. The rest of the sequences is automatically aligned by DIALIGN given the constraints defined by this anchor point. Although only one single column has been used for anchoring, the tree blocks are almost perfectly aligned.

As an example, Figure 4 shows an alignment calculated by the non-anchored default version of DIALIGN for BAliBASE reference set *lr69*. This sequence set consists of four DNA-binding proteins and is a challenging alignment example as there is only weak similarity at the primary sequence level. These proteins contain three *core blocks *for which a reliable multi-alignment is known based on 3D-structure information. As shown in Figure 4, most of the core blocks are misaligned by DIALIGN because of the low level of sequence similarity. With the BAliBASE scoring system for multiple alignments, the default alignment produced by DIALIGN has a *sum-of-pairs score *of only 33%, i.e. 33% of the amino-acid pairs in the core blocks are correctly aligned. The *column score *of this alignment 0%, i.e. there is not a single column of the core blocks correctly aligned.

We investigated how many anchor points were necessary to enforce a correct alignment of the three core blocks in this test example. As it turned out, it was sufficient to use one single column of the core blocks as anchor points, namely the first column of the third motif. Technically, this can be done by using three anchor points of length one each: anchor point connecting the first position of this core block in sequence 1 with the corresponding position in sequence 2, another anchor connecting sequence 1 with sequence 3 and a third anchor connecting sequence 1 with sequence 4. Although our anchor points enforced the correct alignment only for a single column, most parts of the core blocks were correctly aligned as shown in Figure 4. The BAliBASE sum-of-pairs score of the resulting alignment was 91% while the column score was 90% as 18 out of 20 columns of the core blocks were correctly aligned. As was generally the case for BAliBASE, the *DIALIGN score *of the (biologically meaningful) anchored alignment was lower than the score of the (biologically wrong) default alignment. The DIALIGN score of the anchored alignment was 9.82 compared with 11.99 for the non-anchored alignment, so here the score of the anchored alignment was around 18 percent below the score of the non-anchored alignment.

## Anchored alignments for phylogenetic footprinting

Evolutionarily conserved regions in non-coding sequences represent a potentially rich source for the discovery of gene regulatory regions. While functional elements are subject to stabilizing selection, the adjacent non-functional DNA evolves much faster. Therefore, blocks of conservation, so-called phylogenetic footprints, can be detected in orthologous non-coding sequences with low overall similarity by comparative genomics [39]. Alignment algorithms, including DIALIGN, were advocated for this task. As the example in the previous section shows, however, anchoring the alignments becomes a necessity in applications to large genomic regions and clusters of paralogous genes. While interspersed repeats are normally removed ("masked") using e.g. *RepeatMasker*, they need to be taken into account in the context of phylogenetic footprinting: if a sequence motif is conserved hundreds of millions of years it may well have become a regulatory region even if it is (similar to) a repetitive sequence in some of the organisms under consideration [40].

The phylogenetic footprinting program *TRACKER *[41] was designed specifically to search for conserved non-coding sequences in large gene clusters. It is based on a similar philosophy as segment based alignment algorithms. The TRACKER program computes pairwise local alignments of all input sequences using BLASTZ [42] with non-stringent settings. BLASTZ permits alignment of long genomic sequences with large proportions of neutrally evolving regions. A post-processing step aims to remove simple repeats recognized at their low sequence complexity and regions of low conservation. The resulting list of pairwise alignments is then assembled into clusters of partially overlapping regions. Here the approach suffers from the same problem as DIALIGN, which is, however, resolved in a different way: instead of producing a single locally optimal alignment, TRACKER lists all maximal compatible sets of pairwise alignments. For the case of Figure 1(C), for instance, we obtain both *M*_2_*M*_3 _and *M*_2_*M*_3_. Since this step is performed based on the overlap of sequence intervals without explicitly considering the sequence information at all, TRACKER is very fast as long as the number of conflicting pairwise alignments remains small. In the final step DIALIGN is used to explicitly calculate the multiple sequence alignments from the subsequences that belong to individual clusters.

For the initial pairwise local alignment step the search space is restricted to orthologous intergenic regions, parallel strands and chaining hits. Effectively, TRACKER thus computes alignments anchored at the genes from BLASTZ fragments.

We have noticed [43] that DIALIGN is more sensitive than TRACKER in general. This is due to detection of smaller and less significant fragments with DIALIGN compared to the larger, contiguous fragments returned by BLASTZ. The combination of BLASTZ and an anchored version of DIALIGN appears to be a very promising approach for phylogenetic footprinting. It makes use of the alignment specificity of BLASTZ and the sensitivity of DIALIGN. A combination of anchoring at appropriate genes (with maximal weight) and BLASTZ hits (with smaller weights proportional e.g. to – log *E *values) reduces the CPU requirements for the DIALIGN alignment by more than an order of magnitude. While this is still much slower than TRACKER (20 min vs. 40 s) it increases the sensitivity of the approach by about 30 – 40% in the Fugu example, Table 1. Work in progress aims at improving the significance measures for local multiple alignments. A more thorough discussion of anchored segment-based alignments to phylogenetic footprinting will be published elsewhere.

## Conclusion

Automated alignment procedures are based on simple algorithmical rules. For a given set of input sequences, they try to find an alignment with maximum score in the sense of some underlying objective function. The two basic questions in sequence alignment are therefore (*a*) to define an meaningful objective function and (*b*) to design an efficient optimisation algorithm that finds optimal or at least near-optimal alignments with respect to the chosen objective function. Most multi-alignment programs are using *heuristic *optimisation algorithms, i.e. they are, in general, not able to find the mathematically optimal alignment with respect to the objective function. An objective function for sequence alignment should assign *numerically *high scores to *biologically *meaningful alignments. However, it is clearly not possible to find a *universally *applicable objective function that would give highest numerical scores to the biologically correct alignments in all possible situations. This is the main reason why alignment programs may fail to produce biologically reasonable output alignments. In fact, the impossibility to define a universal objective function constitutes a fundamental limitation for *all *automated alignment algorithms.

Often a user is already familiar with a sequence family that he or she wants to align, so some knowledge about existing sequence homologies may be available. Such expert knowledge can be used to direct an otherwise automated alignment procedure. To facilitate the use of expert knowledge for sequence alignment, we proposed an *anchored alignment *approach where known homologies can be used to restrict the alignment search space. This can clearly improve the quality of the produced output alignments in situations where automatic procedures are not able to produce meaningful alignments. In addition, alignment anchors can be used to reduce the program running time. For the *Hox *gene clusters that we analyzed, the non-anchored version of DIALIGN produced serious misalignments. We used the known gene boundaries as anchor points to guarantee a correct alignment of these genes to each other.

There are two possible reasons why automated alignment procedures may fail to produce biologically correct alignments, (*a*) The chosen objective function may not be in accordance with biology, i.e., it may assign mathematically high scores to biologically wrong alignments. In this case, even efficient optimisation algorithms would lead to meaningless alignments. (*b*) The mathematically optimal alignment is biologically meaningful, but the employed heuristic optimisation procedure is not able to find the alignment with highest score. For the further development of alignment algorithms, it is crucial to find out which one of these reasons is to blame for mis-alignments produced by existing software programs. If (*a*) is often observed for an alignment program, efforts should be made to improve its underlying objective function. If (*b*) is the case, the biological quality of the output alignments can be improved by using a more efficient optimisation algorithm. For DIALIGN, it is unknown how close the produced alignments come to the numerically optimal alignment – in fact, it is possible to construct example sequences where DIALIGN's greedy heuristic produces alignments with arbitrarily low scores compared with the possible optimal alignment.

In the Fugu example, Figure 2 and 3, the *numerical *alignment score of the (anchored) correct alignment was 13% below the score of the non-anchored alignment. All sequences in Figure 2 and 3 contain only subsets of the 13 *Hox *paralogy groups, and different sequences contain different genes. For such an extreme data set, it is unlikely that any reasonable objective function would assign an optimal score to the biologically correct alignment. Here, the problem is that sequence similarity no longer coincides with biological homology. The only way of producing good alignments in such situations is *to force *a program to align certain known homologies to each other. With our anchoring approach we can do this, for example by using known gene boundaries as *anchor points*.

For the BAliBASE benchmark data base, the total score of the (biologically meaningful) anchored alignments was also below the score of the (biologically wrong) non-anchored default alignments.

This implies, that improved optimisation algorithms will not lead to biologically improved alignments for these sequences. In this case, however, there is some correspondence between sequence similarity and homology, so one should hope that the performance of DIALIGN on these data can be improved by to designing better objective functions. An interesting example from BAliBASE is shown in Figure 4. Here, the non-anchored default version of our program produced a complete mis-alignment. However, it was sufficient to enforce the correct alignment of one *single *column using corresponding anchor points to obtain a meaningful alignment of the entire sequences where not only the one anchored column but most of the three core blocks are correctly aligned. This indicates that the correct alignment of the core blocks corresponds to a *local maximum *in the alignment landscape.

In contrast, in the teleost *HoxA *cluster example the numerical score of the anchored alignment was around 15% *above *the score of the non-anchored alignment. This demonstrates that the greedy optimisation algorithm used by DIALIGN can lead to results with scores far below the optimal alignment. In such situations, improved optimisation algorithms may lead not only to mathematically higher-scoring alignments but also to alignments that are closer to the biologically correct alignment. We will use our anchored-alignment approach systematically to study the efficiency of objective functions and optimisation algorithms for our segment-based approach to multiple sequence alignment.

## Program availability

The program is available online and as downloadable source code at Göttingen Bioinformatics Compute Server (GOBICS) [44].

## Competing interests

The author(s) declare that they have no competing interests.
